# Cross-Sectional Survey of Smoking Patterns During the COVID-19 Pandemic in a Tobacco Cessation and Lung Cancer Screening Program

**DOI:** 10.31486/toj.21.0082

**Published:** 2022

**Authors:** Maria C. Mejia, Roger Zoorob, Robert S. Levine, Xiaofan Huang, Charles H. Hennekens

**Affiliations:** ^1^ Department of Family and Community Medicine, Baylor College of Medicine, Houston, TX; ^2^ Charles E. Schmidt College of Medicine, Florida Atlantic University, Boca Raton, FL; ^3^ Dan L. Duncan Institute for Clinical and Translational Research, Baylor College of Medicine, Houston, TX

**Keywords:** *COVID-19*, *smoking*, *smoking cessation*, *tobacco cessation*, *tobacco smoking*

## Abstract

**Background:** Coronavirus disease 2019 (COVID-19) produces a wide array of deleterious consequences, some of which are unintended. Data are sparse on whether, and if so, how, current cigarette smoking habits are affected by COVID-19. We describe changes to smoking habits and their correlates during the COVID-19 pandemic among participants in a tobacco cessation and lung cancer screening program.

**Methods:** Between June and October 2020, we conducted a cross-sectional survey of a convenience sample of 150 participants in a lung cancer screening and tobacco cessation program. The survey consisted of 3 parts: (1) changes in tobacco use, (2) impact and coping strategies toward COVID-19, and (3) COVID-19 exposure and use of protective measures. Demographic variables included age, sex, race/ethnicity, and marital status.

**Results:** All 150 participants who were contacted agreed to participate in this cross-sectional survey. The statistically significant correlates of increased tobacco use were high uncertainty about the future (*P*<0.001), loneliness because of social distancing or self-isolating (*P*<0.001), anger or frustration with how the pandemic has disrupted daily life (*P*<0.001), boredom resulting from inability to work or engage in regular daily activities/routines (*P*<0.001), desire to cope using alcohol or drugs (*P*=0.002), sadness or feelings of hopelessness (*P*=0.003), and worry or fear about challenges to securing basic needs such as groceries or medication (*P*<0.001). In contrast, those who smoked less were more likely to practice social distancing (*P*=0.002) and use protective measures (*P*=0.005).

**Conclusion:** Among those who decreased or stopped smoking, correlates included greater use of protective measures for COVID-19, including social distancing and testing. These data may aid healthcare providers to identify and provide counsel to cigarette smokers at greater risks for increasing tobacco consumption during stresses such as COVID-19.

## INTRODUCTION

Since 1965, the prevalence of cigarette smoking has markedly decreased in the United States.^1^ Despite the remarkable decline, cigarette smoking causes hundreds of thousands of potentially avoidable premature deaths in the United States each year, principally from lung cancer, cardiovascular disease, and chronic respiratory disease.^1^ While coronavirus disease 2019 (COVID-19) has caused more than 944,000 premature deaths since January 2020, the US Centers for Disease Control and Prevention estimates that tobacco-related causes account for more than 480,000 deaths per year in the United States.^1,2^

Tobacco consumption among US smokers has increased by more than 30% during the COVID-19 pandemic.^3^ COVID-19 has also adversely impacted screening,^4^ diagnosis,^5^ and survival from lung cancer.^6^ Moreover, concerns have been raised about adverse effects of the COVID-19 pandemic on smokers trying to quit in the United States.^7^

To the best of our knowledge, however, data are sparse about the possible interrelationships and characteristics of cigarette smokers who have increased and decreased their habits during the COVID-19 pandemic. In this report, we describe changes to smoking habits and correlates of increases and decreases during the COVID-19 pandemic, including key indicators of psychological distress among participants enrolled in a tobacco cessation and lung cancer screening program.

## METHODS

### Design

Between June and October 2020, we conducted a cross-sectional survey of a convenience sample of 150 participants in a lung cancer screening and tobacco cessation program. Participants were eligible for the program if they were 55 to 80 years of age, had a tobacco history of at least 30 pack-years, and were currently smoking or had quit smoking within the prior 15 years.^8^ The program involved 6 to 8 visits, including an initial face-to-face contact lasting 45 to 60 minutes and follow-up in person or by phone. As the US COVID-19 epidemic increased markedly, all visits transitioned to phone contacts, and educational materials were mailed to participants along with nicotine replacement therapies if indicated. Each participant was asked about participating in the survey during regular phone follow-up until 150 participants were enrolled. Data were collected by 3 trained interviewers whose backgrounds included fact-checking and verification.

Our sample size of 150 provided sufficient statistical power to detect small to moderate differences in smoking habits.^9^ Findings are reported in accordance with the Strengthening of the Reporting of Observational Studies in Epidemiology (STROBE) guidelines.^10^ The study was approved by the Institutional Review Board of the Baylor College of Medicine.

### Measures

The survey consisted of 3 parts: (1) changes in tobacco use, (2) impact and coping strategies toward COVID-19, and (3) COVID-19 exposure and use of protective measures. Demographic variables included age, sex, race/ethnicity, and marital status. The survey (Appendix) included 28 questions adapted from the CoRonavIruS Health Impact Survey^11^ and the US Centers for Disease and Control Prevention guidance for COVID-19.^12^ Tobacco use change during the COVID-19 pandemic was classified as increased, unchanged, decreased, varied day to day with no clear pattern, and not applicable/former smoker. Coping strategies and mental health questions were answered on a 5-point Likert scale from least (1) to greatest (5). We included 4 questions about the clinical symptoms of COVID-19 and 3 questions about protective strategies. Five questions referred to mitigation practices, also scored on a 5-point Likert scale.

### Data Analysis

We first explored whether significant differences were found among those who increased, decreased, or continued their same smoking patterns. Categorical variables are summarized by frequencies and percentages. Continuous variables are summarized by medians with interquartile ranges. We stratified the data by changes in smoking use overall and by race/ethnicity and tested for statistical significance using Kruskal-Wallis or chi-squared tests. To explore interrelationships of changes in smoking with categorical baseline characteristics, we used paired chi-squared tests with Holm *P* value adjustments to explore which smoking use change and race/ethnicity group were significantly different from other categories. To explore continuous variables, we used pairwise Wilcoxon rank sum tests with Holm *P* value adjustments. We considered 2-sided *P* values at <0.05 as significant.

## RESULTS

**Figure 1.**
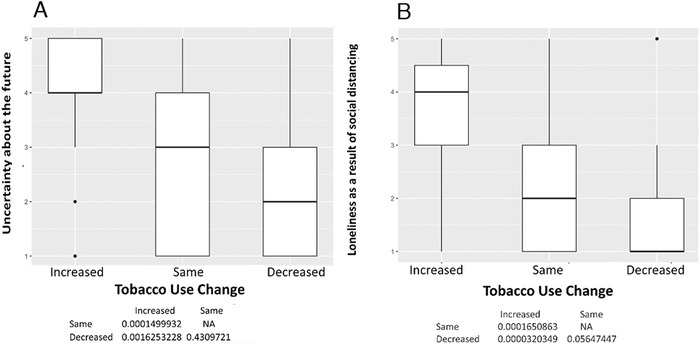
**(A) Uncertainty about the future. Participants with increased tobacco use had more uncertainty about the future compared to those with unchanged (median 4 vs 3, *P*<0.001) or decreased tobacco use (median 4 vs 2, *P*=0.002). (B) Loneliness as a result of social distancing. Participants with increased tobacco use were more likely to report loneliness as a result of social distancing compared to those with unchanged (median 4 vs 2, *P*<0.001) or decreased tobacco use (median 4 vs 1, *P*<0.001).** NA, not applicable.

**Figure 2.**
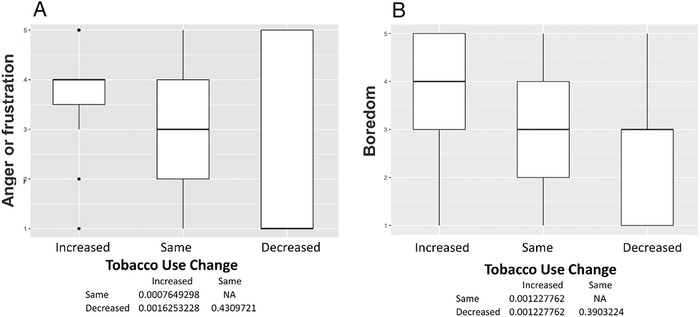
**(A) Anger or frustration with how the pandemic has disrupted your daily life. Participants with increased tobacco use reported more anger or frustration with how the pandemic has disrupted their daily life compared to those with unchanged tobacco use (median 4 vs 3, *P*<0.001). (B) Boredom due to being unable to work or engage in regular daily activities/routines. Participants with increased tobacco use reported more boredom compared to those with unchanged (median 4 vs 3, *P*=0.001) or decreased tobacco use (median 4 vs 3, *P*=0.001).** NA, not applicable.

**Figure 3.**
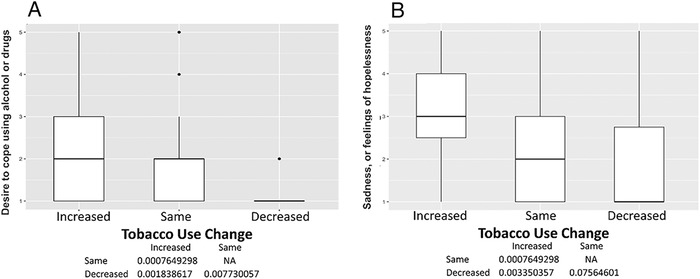
**(A) Desire to cope using alcohol or drugs. Participants with decreased tobacco use had less desire to cope using alcohol or drugs compared to those with unchanged (median 1 vs 2, *P*=0.008) or increased tobacco use (median 1 vs 2, *P*=0.002). (B) Sadness, or feelings of hopelessness. Participants with increased tobacco use had more sadness or feelings of hopelessness compared to those with unchanged (median 3 vs 2, *P*=0.006) or decreased tobacco use (median 3 vs 1, *P*=0.003).** NA, not applicable.

**Figure 4.**
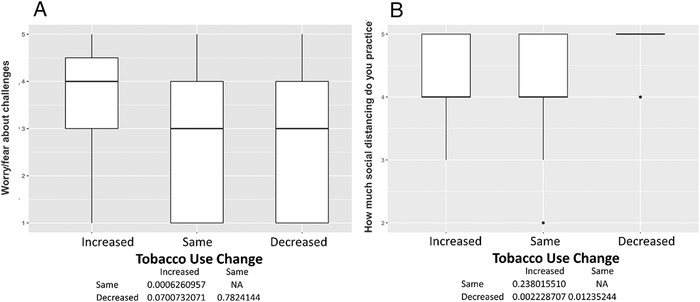
**(A) Worry/fear about challenges to securing basic needs such as groceries or medication. Participants with decreased tobacco use had more worry or fear about challenges to securing basic needs compared to those with unchanged tobacco use (median 4 vs 3, *P*<0.001). (B) How much social distancing do you practice? Participants with decreased tobacco use practiced more social distancing than those with unchanged (median 5 vs 4, *P*=0.012) or increased tobacco use (median 5 vs 4, *P*=0.002).** NA, not applicable.

**Figure 5.**
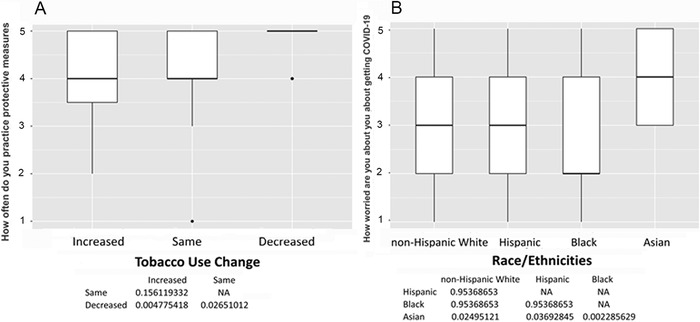
**(A) How often do you practice protective measures such as hand washing, disinfecting household surfaces, using hand sanitizer? Participants with decreased tobacco use practiced protective measures more often than those with unchanged (median 5 vs 4, *P*=0.027) or increased tobacco use (median 5 vs 4, *P*=0.005). (B) How worried are you about you or individuals in your household getting COVID-19? Participants who were Asian worried more about themselves or individuals in their household getting COVID-19 compared to patients who were non-Hispanic White (median 4 vs 3, *P*=0.025), Hispanic (median 4 vs 3, *P*=0.037), or Black (median 4 vs 2, *P*=0.002).** NA, not applicable.

Among the 150 participants, 50 (33%) reported changes in tobacco use, 60 (40%) reported no changes, and 40 (27%) were former smokers. No former smokers reported relapsing. The mean age was 60.7 ± 4.5 years, and 97 participants (65%) were male (Table 1). Participants who reported increases in smoking had high levels of uncertainty about the future (*P*<0.001) (Figure 1A), loneliness as a result of social distancing or self-isolating (*P*<0.001) (Figure 1B), anger or frustration with how the pandemic has disrupted daily life (*P*<0.001) (Figure 2A), boredom because of being unable to work or engage in regular daily activities/routines (*P*=0.001) (Figure 2B), a desire to cope using alcohol or drugs (*P*=0.002) (Figure 3A), sadness or feelings of hopelessness (*P*=0.003) (Figure 3B), and worry or fear about challenges to securing basic needs such as groceries or medication (*P*<0.001) (Figure 4A). In contrast, those who smoked less were more likely to practice social distancing (*P*=0.002) (Figure 4B) and practice other protective measures (*P*=0.005) (Figure 5A).

**Table 1.** Participant Demographics, n=150

Variable	Value
Age, years, mean ± SD	60.7 ± 4.5
Sex	
Female	53 (35)
Male	97 (65)
Race	
Black	73 (49)
White	64 (43)
Asian/Pacific Islander	13 (9)
Ethnicity	
Non-Hispanic	117 (78)
Hispanic	33 (22)
Marital status^a^	
Single	72 (51)
Married	41(29)
Divorced	24 (17)
Widowed	5 (4)

Note: Data are presented as n (%) unless otherwise indicated.

^a^n=142.

Table 2 shows survey responses stratified by tobacco usage. The numbers of participants tested for COVID-19 were significantly different between the groups (*P*=0.022). We found no statistically significant difference between tobacco use status and current housing, whether the participant had perceived control over contracting COVID-19, or source of information about COVID-19.

**Table 2.** Survey Questions Stratified by Tobacco Usage, n=110

	Change in Tobacco Usage			
Survey Question	Increased, n=31	Stayed the Same, n=60	Decreased, n=19	*P* Value
Tobacco usage has changed during the COVID-19 pandemic, n (%)	31 (100.0)	0 (0.0)	19 (100.0)	<0.001
COVID-19 pandemic has affected your desire to participate in our LDCT lung cancer screening program, n (%)	4 (12.9)	3 (5.0)	2 (10.5)	0.393
**Coping strategies and mental health, median [IQR]**				
Uncertainty about the future	4.00 [4.00, 5.00]	3.00 [1.00, 4.00]	2.00 [1.00, 3.00]	<0.001
Loneliness as a result of social distancing/self-isolating	4.00 [3.00, 4.50]	2.00 [1.00, 3.00]	1.00 [1.00, 2.00]	<0.001
Anger or frustration with how the pandemic has disrupted your daily life	4.00 [3.50, 4.00]	3.00 [2.00, 4.00]	1.00 [1.00, 5.00]	<0.001
Boredom due to being unable to work or engage in regular daily activities/routines	4.00 [3.00, 5.00]	3.00 [2.00, 4.00]	3.00 [1.00, 3.00]	0.001
Desire to cope using alcohol or drugs	2.00 [1.00, 3.00]	2.00 [1.00, 2.00]	1.00 [1.00, 1.00]	0.002
Sadness or feelings of hopelessness	3.00 [2.50, 4.00]	2.00 [1.00, 3.00]	1.00 [1.00, 2.75]	0.003
Worry or fear about challenges to securing basic needs such as groceries or medication	4.00 [3.00, 4.50]	3.00 [1.00, 4.00]	3.00 [1.00, 4.00]	<0.001
**Health management, n (%)**				
Have a serious or chronic health condition that requires medication and management at home	12 (38.7)	20 (33.3)	12 (63.2)	0.068
**Effects on housing and employment, n (%)**				
I live alone	7 (22.6)	18 (30.0)	5 (26.3)	0.749
I live with spouse/significant other	11 (35.5)	21 (35.0)	9 (47.4)	0.634
I live with another family member	7 (22.6)	16 (26.7)	4 (21.1)	0.846
I live with a non-family member	6 (19.4)	5 (8.3)	1 (5.3)	0.191
COVID-19 affected my housing situation	6 (19.4)	5 (8.3)	1 (5.3)	0.191
COVID-19 affected my employment status	9 (29.0)	15 (25.0)	5 (26.3)	0.911
**Exposure and preventive measures**				
COVID-19 symptoms in the past 2 weeks, n (%)				
Fever	0 (0.0)	1 (1.7)	0 (0.0)	0.657
Dry cough	0 (0.0)	2 (3.3)	0 (0.0)	0.428
Runny nose	0 (0.0)	1 (1.7)	0 (0.0)	0.657
Body aches	0 (0.0)	1 (1.7)	1 (5.3)	0.398
Upset stomach	0 (0.0)	0 (0.0)	0 (0.0)	NA
Loss of smell/taste	0 (0.0)	0 (0.0)	0 (0.0)	NA
Fatigue, tiredness	1 (3.2)	1 (1.7)	1 (5.3)	0.689
None of the above	30 (96.8)	56 (93.3)	18 (94.7)	0.790
Had close contact with an individual with flu-like symptoms or a confirmed diagnosis of COVID-19 in the past 3 weeks, n (%)				
Yes	2 (6.5)	1 (1.7)	0 (0.0)	
No	27 (87.1)	55 (91.7)	16 (84.2)	
Unsure	2 (6.5)	4 (6.7)	3 (15.8)	
Have been tested for COVID-19, n (%)	4 (12.9)	14 (23.3)	9 (47.4)	0.022
Believed might have COVID-19 but did not get tested, n (%)	1 (3.2)	5 (8.3)	0 (0.0)	0.662
Reside in area under a “shelter-in-place” or “stay-at-home” order, n (%)	31 (100.0)	59 (98.3)	18 (94.7)	–
Avoided crowded places whenever possible, n (%)	30 (96.8)	58 (96.7)	19 (100.0)	0.724
Wore a mask when leaving the house, n (%)	30 (96.8)	60 (100.0)	19 (100.0)	0.276
Worried about you or individuals in your household getting COVID-19, median [IQR]	4.00 [2.00, 4.00]	2.50 [2.00, 4.00]	2.00 [1.00, 5.00]	0.081
Practice social distancing, median [IQR]	4.00 [4.00, 5.00]	4.00 [4.00, 5.00]	5.00 [5.00, 5.00]	0.002
Practice protective measures such as hand washing, disinfecting household surfaces, using hand sanitizer, median [IQR]	4.00 [3.50, 5.00]	4.00 [4.00, 5.00]	5.00 [5.00, 5.00]	0.005
Control you feel like you have over whether or not you or household might contract COVID-19, median [IQR]	3.00 [3.00, 4.00]	4.00 [3.00, 5.00]	4.00 [3.00, 5.00]	0.094
Feel equipped with enough knowledge to protect yourself and your household from COVID-19, median [IQR]	3.00 [3.00, 4.00]	4.00 [3.00, 5.00]	4.5 [3.25, 5.00]	0.064
**Source of COVID-19 information and updates, n (%)**				
National news outlet (FOX, CNN, MSNBC, etc.)	20 (64.5)	37 (61.7)	13 (68.4)	0.861
Local news on television	27 (87.1)	53 (88.3)	17 (89.5)	0.967
Newspaper or local print media	1 (3.2)	3 (5.0)	2 (10.5)	0.530
Directly from family or friends	8 (25.8)	14 (23.3)	9 (47.4)	0.120
A healthcare provider	2 (6.5)	6 (10.0)	4 (21.1)	0.260
Social media (Facebook, Instagram, Twitter)	3 (9.7)	12 (20.0)	5 (26.3)	0.289
Messaging platform (WhatsApp, Facebook)	0 (0.0)	1 (1.7)	2 (10.5)	0.065
Other	2 (6.5)	7 (11.7)	1 (5.3)	0.583

Note: Responses reported as median [interquartile range (IQR)] were scored according to a Likert scale ranging from 1 (least) to 5 (greatest).

COVID-19, coronavirus disease 2019; LDCT, low dose computed tomography.

Table 3 shows survey responses stratified by race/ethnicity. Among the 150 participants, 48.7% were Black, 20.7% were non-Hispanic White, 22% were Hispanic, and 8.7% were Asian/Pacific Islander. We found significant differences by race/ethnicity in the effect of COVID-19 on employment status (*P*=0.042). The levels of “How worried are you about you or individuals in your household getting COVID-19?” differed significantly by race/ethnicity (*P*=0.004), and Figure 5B shows that Asian participants were more worried than the participants in other racial/ethnic groups. The number of Hispanic participants who avoided crowded places was significantly lower than the other groups (*P*=0.029). Compared to other groups, a significantly lower number of Hispanic participants received COVID-19 information from a national news outlet (*P*=0.033), and more Hispanics received information directly from family or friends (*P*=0.003). We found no statistically significant differences by race/ethnicity with respect to change in tobacco usage, desire to participate in the screening/tobacco control program, uncertainty about the future, loneliness, anger or frustration about how the pandemic has disrupted daily life, boredom, desire to cope with alcohol or drugs, feelings of sadness or hopelessness, or worry or fear about challenges to securing basic needs.

**Table 3.** Survey Questions Stratified by Race/Ethnicity, n=150

	**Race/Ethnicity**				
Survey Question	Non-Hispanic White, n=31	Hispanic, n=33	Black, n=73	Asian/Pacific Islander, n=13	*P* Value
Tobacco usage has changed during the pandemic, n (%)	12 (38.7)	9 (27.3)	25 (34.2)	4 (30.8)	0.600
Way your tobacco usage changed during the COVID-19 pandemic, n (%)	n=23	n=20	n=58	n=9	0.748
Increased	8 (34.8)	5 (25.0)	17 (29.3)	1 (11.1)	
Decreased	4 (17.4)	4 (20.0)	8 (13.8)	3 (33.3)	
Stayed the same	11 (47.8)	11 (55.0)	33 (56.9)	5 (55.6)	
COVID-19 pandemic has affected your desire to participate in our LDCT lung cancer screening program, n (%)	2 (6.5)	2 (6.1)	8 (11.0)	0 (0.0)	0.519
**Coping strategies and mental health, median [IQR]**					
Uncertainty about the future	3.00 [3.00, 4.00]	4.00 [2.00, 5.00]	3.00 [1.00, 4.00]	4.00 [2.00, 4.00]	0.467
Loneliness as a result of social distancing/self-isolating	3.00 [2.00, 4.00]	3.00 [2.00, 3.00]	2.00 [1.00, 4.00]	2.00 [1.00, 3.00]	0.484
Anger or frustration with how the pandemic has disrupted your daily life	3.00 [2.00, 4.00]	4.00 [2.00, 4.00]	3.00 [2.00, 4.00]	3.00 [2.00, 3.00]	0.489
Boredom due to being unable to work or engage in regular daily activities/routines	3.00 [2.00, 4.00]	3.00 [2.00, 4.00]	3.00 [2.00, 4.00]	3.00 [2.00, 3.00]	0.855
Desire to cope using alcohol or drugs	1.00 [1.00, 2.00]	1.00 [1.00, 2.00]	1.00 [1.00, 2.00]	1.00 [1.00, 1.00]	0.172
Sadness or feelings of hopelessness	2.00 [2.00, 3.00]	2.00 [1.00, 3.00]	2.00 [1.00, 3.00]	2.00 [1.75, 4.25]	0.839
Worry or fear about challenges to securing basic needs such as groceries or medication	3.00 [2.00, 4.00]	4.00 [1.00, 4.00]	3.00 [2.00, 4.00]	3.00[3.00, 4.00]	0.901
**Health management, n (%)**					
Have a serious or chronic health condition that requires medication and management at home	13 (41.9)	9 (27.3)	30 (41.1)	3 (23.1)	0.351
**Effects on housing and employment, n (%)**					
I live alone	10 (32.3)	4 (12.1)	31 (42.5)	0 (0.0)	0.001
I live with spouse/significant other	11 (35.5)	20 (60.6)	19 (26.0)	11 (84.6)	<0.001
I live with another family member	6 (19.4)	7 (21.2)	17 (23.3)	2 (15.4)	0.916
I live with a non-family member	4 (12.9)	2 (6.1)	6 (8.2)	0 (0.0)	0.509
COVID-19 affected my housing situation	4 (12.9)	2 (6.1)	9 (12.5)	1 (7.7)	0.738
COVID-19 affected my employment status	5 (16.1)	10 (30.3)	15 (20.5)	7 (53.8)	0.042
**Exposure and preventive measures**					
COVID-19 symptoms in the past 2 weeks, n (%)					
Fever	0 (0.0)	0 (0.0)	1 (1.4)	0 (0.0)	0.786
Dry cough	0 (0.0)	0 (0.0)	2 (2.7)	0 (0.0)	0.544
Runny nose	0 (0.0)	0 (0.0)	1 (1.4)	0 (0.0)	0.786
Body aches	0 (0.0)	0 (0.0)	2 (2.7)	0 (0.0)	0.544
Upset stomach	0 (0.0)	0 (0.0)	1 (1.4)	0 (0.0)	0.786
Loss of smell/taste	0 (0.0)	0 (0.0)	0 (0.0)	0 (0.0)	N/A
Fatigue, tiredness	0 (0.0)	0 (0.0)	3 (4.1)	0 (0.0)	0.358
None of the above	31 (100.0)	33 (100.0)	66 (90.4)	13 (100.0)	0.052
Had close contact with an individual with flu-like symptoms or a confirmed diagnosis of COVID-19 in the past 3 weeks, n (%)					0.816
Yes	1 (3.2)	1 (3.0)	3 (4.1)	0 (0.0)	
No	26 (83.9)	31 (93.9)	62 (84.9)	12 (92.3)	
Unsure	4 (12.9)	1 (3.0)	8 (11.0)	1 (7.7)	
Have been tested for COVID-19, n (%)	7 (22.6)	4 (12.1)	23 (31.5)	2 (15.4)	0.147
Believed might have had COVID-19 but did not get tested, n (%)	2 (6.5)	2 (6.1)	3 (4.1)	0 (0.0)	0.786
Reside in an area under a “shelter-in-place” or “stay-at-home” order, n (%)	30 (96.8)	33 (100.0)	70 (95.9)	13 (100.0)	–
Avoided crowded places whenever possible, n (%)	30 (96.8)	28 (84.8)	72 (98.6)	12 (92.3)	0.029
Wore a mask when leaving the house, n (%)	31 (100.0)	32 (97.0)	73 (100.0)	13 (100.0)	0.312
Worried about you or individuals in your household getting COVID-19, median [IQR]	3.00 [2.00, 4.00]	3.00 [2.00, 4.00]	2.00 [2.00, 4.00]	4.00 [3.00, 5.00]	0.004
Practice social distancing, median [IQR]	5.00 [4.00, 5.00]	4.00 [4.00, 5.00]	5.00 [4.00, 5.00]	4.00 [4.00, 5.00]	0.602
Practice protective measures such as hand washing, disinfecting household surfaces, using hand sanitizer, median [IQR]	5.00 [4.00, 5.00]	4.00 [4.00, 5.00]	5.00 [4.00, 5.00]	4.00 [4.00, 5.00]	0.677
Control you feel you have over whether or not you or household might contract COVID-19, median [IQR]	4.00 [3.00, 4.00]	3.00 [2.00, 4.00]	4.00 [3.00, 4.00]	3.00 [3.00, 4.00]	0.394
Feel equipped with enough knowledge to protect yourself and your household from COVID-19, median [IQR]	4.00 [3.00, 4.00]	3.00 [3.00, 4.00]	4.00 [3.00, 5.00]	3.50 [3.00, 5.00]	0.068
**Source of COVID-19 information and updates, n (%)**					
National news outlet (FOX, CNN, MSNBC, etc.)	22 (71.0)	15 (45.5)	54 (74.0)	9 (69.2)	0.033
Local news on television	30 (96.8)	31 (93.9)	62 (84.9)	11 (84.6)	0.229
Newspaper or local print media	3 (9.7)	2 (6.1)	2 (2.7)	0 (0.0)	0.371
Directly from family or friends	5 (16.1)	16 (48.5)	13 (17.8)	5 (38.5)	0.003
A healthcare provider	2 (6.5)	4 (12.1)	9 (12.3)	1 (7.7)	0.805
Social media (Facebook, Instagram, Twitter)	7 (22.6)	4 (12.1)	9 (12.3)	2 (15.4)	0.563
Messaging platform (WhatsApp, Facebook)	0 (0.0)	2 (6.1)	2 (2.7)	0 (0.0)	0.445
Other	3 (9.7)	1 (3.0)	6 (8.2)	3 (23.1)	0.188

Note: Responses reported as median [interquartile range (IQR)] were scored according to a Likert scale ranging from 1 (least) to 5 (greatest).

COVID-19, coronavirus disease 2019; LDCT, low dose computed tomography.

## DISCUSSION

Among 150 participants in a tobacco cessation and lung cancer screening program during the COVID-19 pandemic, we found statistically significant and potentially clinically important differences between those who increased and decreased tobacco use. Among current smokers, 28.2% (31/110) reported increased tobacco use, 17.3% (19/110) decreased, and 54.5% (60/110) no change. In addition, there were no reports of relapse among former smokers.

We found correlates of increased tobacco use related to coping strategies and mental health such as high uncertainty about the future, loneliness as a result of social distancing, anger or frustration with how the pandemic has disrupted daily life, boredom because of being unable to work or engage in regular daily activities/routines, desire to cope using alcohol or drugs, sadness or feelings of hopelessness, and worry or fear about challenges to securing basic needs such as groceries or medication. In contrast, those who smoked less were more likely to practice social distancing and other protective measures.

The current data are compatible with previous reports of a bidirectional effect of COVID-19 observed in smokers: a reduction in or cessation of smoking in some but an increase in others.^13,14^ In an online survey of 1,491 adults in Australia, all aspects of psychological distress—specifically depression, anxiety, and stress—were significantly correlated with health behavior.^15^ Among 172 smokers completing the survey, 49.9% reported an increase in smoking behavior, while 16.3% reported a reduction.^15^ An online survey of 4,005 French adults during COVID-19 included 1,062 regular smokers; 231 (21.8%) reported that they increased their intake, while 177 (16.7%) reportedly decreased intake.^16^ The study authors suggested that the threat of contracting COVID-19 may have motivated some smokers to improve, while boredom and restrictions in movement may have had the opposite effect in others. In a survey from Italy, smokers reported increased tobacco use, increased food intake, and changes (positive and negative) in sleep quality during home confinement.^17^ High stress levels and the need to adapt to prolonged stays at home, particularly among fully employed individuals, have had significant correlations during COVID-19.^18^

Smoking cessation programs struggle to achieve results. A report by Lang and Yakhkind showed that while the pandemic provides an opportunity to adapt and expand smoking cessation services, potential challenges involve the increased need for behaviorists because of widespread stress brought on, in part, by isolation from friends and family.^19^ Some smokers may continue to smoke because of their erroneous belief that tobacco is a protective factor against COVID-19.^19^ Such erroneous beliefs may lead to decreases in enrollment in smoking cessation programs.^14^

In a survey of smokers in Australia and the United Kingdom, 45% of respondents wanted more information about smoking and COVID-19 risk.^20^ The most popular sources of information identified by respondents were government departments (59%) and physicians (47%), with the preferred delivery platforms being television (61%), online news sources (36%), social media (31%), and e-mail (31%).^20^ This finding contrasts with the data from our study that indicates local and national news outlets were the most common sources of information about the pandemic for respondents, with Hispanics relying more on friends and family members for information than Asians/Pacific Islanders, Blacks, and non-Hispanic Whites.

This study has several limitations that merit consideration. First are the restrictions on generalizability. Participants were tobacco users recruited from a lung cancer screening program who had volunteered to participate in a tobacco cessation program. In addition, all responses were self-reported and may be subject to recall bias. Also, as with any cross-sectional survey, the data are useful to formulate but not test hypotheses.^21,22^

One unique strength of this cross-sectional survey is that all 150 volunteers approached agreed to participate. Another strength is that many hypotheses can be formulated from these descriptive data regarding possible behavioral and other factors that led some individuals to increase and others to decrease their smoking habits during COVID-19. Tests of such hypotheses will require analytic epidemiologic research designed a priori to do so.

## CONCLUSION

These descriptive data show statistically significant differences between those who increased and decreased tobacco use during COVID-19 among volunteers who enrolled in a tobacco cessation and lung cancer screening program in Houston, Texas. Among those who decreased or stopped smoking, correlates included greater use of protective measures for COVID-19 such as social distancing and testing. These data may aid healthcare providers to identify and provide counsel to cigarette smokers at greater risk for increasing tobacco consumption during stresses such as the COVID-19 pandemic.

## Notes

*This article meets the Accreditation Council for Graduate Medical Education and the American Board of Medical Specialties Maintenance of Certification competencies for Patient Care, Medical Knowledge, and Practice-Based Learning and Improvement.*
